# Hepatitis E in Wildlife: Emerging Threats to Human Health

**DOI:** 10.3390/vetsci13020160

**Published:** 2026-02-06

**Authors:** Slavica M. Vesković Moračanin, Branislav I. Kureljušić, Jelena Maletić, Jasna M. Kureljušić, Nemanja V. Jezdimirović, Ana M. Vasić, Bojan Z. Milovanović, Božidar M. Savić

**Affiliations:** 1 Institute of Meat Hygiene and Technology, 11000 Belgrade, Serbia; 2 Institute of Veterinary Medicine of Serbia, 11000 Belgrade, Serbia; branislav.kureljusic@nivs.rs (B.I.K.); jelena.maletic@nivs.rs (J.M.); jasna.kureljusic@nivs.rs (J.M.K.); nemanja.jezdimirovic@nivs.rs (N.V.J.); ana.vasic@nivs.rs (A.M.V.); bojan.milovanovic@nivs.rs (B.Z.M.); bozidar.savic@nivs.rs (B.M.S.)

**Keywords:** hepatitis E virus, wildlife, zoonoses, foodborne pathogens, reservoirs, One Health

## Abstract

Hepatitis E virus (HEV) is a significant public health concern and a leading cause of acute viral hepatitis worldwide. In recent years, HEV has been increasingly recognized as a wildlife-associated zoonotic pathogen, with numerous free-ranging species contributing to its maintenance and transmission. While domestic pigs remain the primary reservoir for human infection globally, growing evidence indicates that wild animals, particularly wild boars, cervids, lagomorphs, rodents, and carnivores, play a critical role in the ecology of HEV. Wild boars are the principal wildlife reservoir, with HEV seroprevalence in Europe ranging from less than 5% to more than 50%, including some of the highest levels reported in the Balkans. In addition to the frequent detection of HEV RNA in wild boar liver and muscle, viral RNA has also been identified in several other game species, most consistently in red deer, roe deer, and, in some regions, hares and wild rabbits, highlighting food safety risks associated with the consumption of raw or undercooked game meat. In regions such as the Balkans, where hunting activities and handling of wild game are widespread, these practices may further increase occupational and dietary exposure to HEV. Rodents may further complicate the epidemiological landscape through environmental contamination of water, soil, and farm surroundings, thereby facilitating indirect transmission pathways. As wildlife populations expand and human–animal interfaces intensify, understanding HEV dynamics in free-ranging species is essential for assessing zoonotic risks and implementing a strengthened One Health approach. This narrative review synthesizes and critically examines current evidence on HEV prevalence, molecular characteristics, and transmission pathways in wildlife, with particular emphasis on Europe and focused consideration of the Balkans as an epidemiologically heterogeneous and underrepresented subregion; examines associated public health implications; and highlights the importance of integrating wildlife into food safety and One Health surveillance frameworks. Existing knowledge gaps and limitations in wildlife surveillance are also discussed.

## Simple Summary

Hepatitis E virus is a cause of liver disease in people and is increasingly recognized as a zoonotic infection that can be shared between animals and humans. While domestic pigs are the main source of human infection, many wild animals also carry the virus and help it persist in nature. This narrative review examines the role of wildlife, especially wild boars, deer, hares, rabbits, rodents, and carnivores, in maintaining and spreading hepatitis E virus. Studies show that wild boars are the most important wildlife source, with high levels of infection reported in several European regions, including the Balkans, which represent an epidemiologically diverse subregion of Europe. The virus has also been found in the meat and organs of other game animals, which means that eating raw or undercooked game meat can pose a risk to people. In areas where hunting and handling of wild game are common, these practices may further increase human exposure to the virus. Small mammals such as rodents may further spread the virus by contaminating the environment. By summarizing current knowledge from Europe, with contextual reference to findings from other parts of the world, this review highlights existing gaps in wildlife surveillance and explains why monitoring wildlife is essential for food safety and public health. Better inclusion of wildlife in disease surveillance can help reduce infection risks and support a coordinated approach to protecting human, animal, and environmental health.

## 1. Introduction

The hepatitis E virus (HEV), named for its enteric transmission route and epidemic potential, is a small, non-enveloped, single-stranded, positive-sense RNA virus classified within the family *Hepeviridae*, genus *Paslahepevirus* [1,2]. It is the primary cause of acute viral hepatitis E and represents a growing yet still underestimated global public health concern that remains underestimated despite increasing recognition. HEV infection is most often asymptomatic or manifests as a self-limiting acute hepatitis; however, in certain populations, particularly pregnant women, immunocompromised individuals, and transplant recipients, the disease may progress to severe and potentially fatal outcomes and may result in chronic hepatitis in a subset of immunocompromised patients, particularly organ transplant recipients [2,3]. In the general population, the overall case fatality rate (CFR) ranges from 0.5% to 3%, but it may reach 25–30% in pregnant women, especially during the third trimester [4]. The clinical picture typically include jaundice, fever, fatigue, abdominal discomfort, and elevated liver enzymes. Beyond hepatic disease, increasing scientific evidence indicates a broader clinical spectrum, with recognized extrahepatic manifestations such as renal impairment, hematological abnormalities, and several neurological syndromes, including Guillain–Barré syndrome, neuralgic amyotrophy, Parsonage–Turner syndrome, and sensory neuropathies, reported in both acute and chronic infections [5,6,7].

Eight genotypes have been described within the species *Paslahepevirus balayani*, according to the most recent ICTV classification [8], of which five (HEV-1 to HEV-4 and HEV-7) are recognized as causative agents of human disease [1]. HEV-1 and HEV-2 infect only humans and are typically associated with large waterborne outbreaks in regions with poor sanitation, particularly in low-income countries [9,10]. Geographically, HEV-1 predominates in Asia and Africa, whereas HEV-2 has been historically reported in Mexico and parts of Africa, with no confirmed human infections documented in recent years. In contrast, HEV-3 and HEV-4 have zoonotic reservoirs in domestic pigs (*Sus scrofa*) and wildlife and account for most autochthonous cases in industrialized countries [2,3]. HEV-3 is globally distributed, while HEV-4 occurs primarily in East Asia and, more recently, has been reported in parts of Europe [3]. HEV-5 and HEV-6 were first identified in wild boar (*Sus scrofa*) in Japan, although their zoonotic potential remains unclear [11]. HEV-7 and HEV-8, detected in dromedary (*Camelus dromedarius*) and Bactrian camels (*Camelus bactrianus*), have demonstrated zoonotic potential, with documented human infection linked to the consumption of camel meat and milk [12,13]. In Europe, genotype 3 remains the predominant cause of human infection, with transmission primarily associated with the consumption of raw or undercooked pork and wild game meat [14].

Globally, HEV is recognized as an emerging public health concern, with serological studies suggesting that nearly one-third of the world’s population has been infected at some point [3,15]. According to the World Health Organization (WHO), HEV caused an estimated 19.47 million acute infections in 2021, resulting in approximately 3450 deaths worldwide [4]. Data from the Centers for Disease Control and Prevention (CDC) further underscore its role as one of the leading causes of acute viral hepatitis, although the true burden is likely underestimated due to the high proportion of asymptomatic or subclinical infections [16]. The European Food Safety Authority (EFSA) Panel on Biological Hazards (BIOHAZ) has also identified HEV as a foodborne pathogen of increasing relevance [17], a finding further supported by recent surveillance data from Europe [14]. In recent years, more than 5000 confirmed human infections have been reported annually. Notably, in January 2024 alone, 520 laboratory-confirmed HEV cases were documented across ten EU/EEA regions [18].

Although the epidemiological landscape varies by region, hepatitis E has clearly transitioned from being regarded as a regional infection to a global health concern, occurring in both developed and developing countries [19,20]. In low-income settings, particularly in parts of Asia and Africa, large outbreaks are predominantly linked to inadequate sanitation and the consumption of contaminated drinking water [4,21]. By contrast, in high-income countries, an increasing number of autochthonous cases are reported, most often associated with zoonotic foodborne transmission through the consumption of raw or undercooked pork, wild boar, and deer meat [14]. Clinical guidelines from the European Association for the Study of the Liver (EASL) further emphasize this dual epidemiological profile, characterized by waterborne outbreaks in low-resource settings and zoonotic, foodborne transmission in industrialized regions [22].

The role of wildlife in the epidemiology of HEV infection is increasingly recognized. Wild boars are the most extensively studied and represent the main reservoir of zoonotic genotypes, particularly HEV-3, in Europe, where prevalence estimates vary considerably between regions and studies, ranging from below 10% to over 30% in certain populations [14,23,24,25]. Growing evidence also highlights the role of cervids, rabbits, and carnivores including wild canids such as red foxes (*Vulpes vulpes*), grey wolves (*Canis lupus*), and martens (*Martes martes*) [26], with several studies suggesting that infections in these species may arise from exposure to rodent-associated viral variants, supporting the “dietary origin hypothesis” [27] within the broader context of HEV genetic diversity and evolution [28]. It should be noted that a clear distinction exists between domestic and wild canids. In domestic dogs, HEV RNA has not been molecularly identified to date, although variable seroprevalence rates have been reported in several European countries. In contrast, wild canids show higher seroprevalence levels, and molecular evidence of HEV infection has been documented in Europe [2,26]. Although rodents are not infected by *Paslahepevirus balayani* (HEV), they may harbour *Rocahepevirus ratti* (ratHEV); sporadic human infections with rat HEV have been reported, but their contribution to the overall burden of hepatitis E remains very limited compared to HEV. Moreover, phylogenetic analyses have demonstrated a high degree of sequence similarity between HEV isolates from humans, domestic pigs, and wildlife, supporting active interspecies transmission [29,30]. These findings emphasize that wild animals play a crucial role in the maintenance and spread of HEV within natural ecosystems, with migration and interactions between domestic and wild populations further increasing the risk of interspecies transmission [23,31]. Accordingly, the epidemiology of HEV must be addressed within the One Health framework, considering the complex interplay between animals, humans, and the environment.

The aim of this paper is to provide a comprehensive overview of the prevalence of HEV in wild animals, with particular emphasis on Europe and the Balkans. It also explores the epidemiological links between wild and domestic animals and their implications for food safety, public health, and veterinary medicine, thereby contributing to a better understanding of the role of wildlife in the transmission of hepatitis E. Special attention is given to preventive and control measures within the One Health framework. This review is intended to serve as a basis for future research and the development of integrated public health and veterinary strategies.

## 2. Materials and Methods

This narrative literature review was conducted using a structured qualitative synthesis approach, focusing on identifying and synthesizing available evidence related to HEV in wildlife and its zoonotic implications. Particular emphasis was placed on epidemiology, transmission routes, molecular characteristics, food safety, and public health risks, all considered within the broader One Health framework. A targeted literature search was carried out in the PubMed, Scopus, Web of Science, and Google Scholar databases, covering the period from 2015 to 2025. The following keywords were used both individually and in combination: Hepatitis E virus, HEV, wildlife, wild boar, deer, rabbits, rodents, foodborne zoonotic pathogens, and One Health. In addition to international peer-reviewed publications, the review also included relevant national academic sources, such as monographs, doctoral dissertations, and official reports approved by the Ministry of Science of the Republic of Serbia, provided they addressed HEV in wildlife.

Study selection was guided by predefined relevance criteria, which are summarized in Table 1.

Inclusion criteria required that studies reported HEV prevalence in wild animal populations, employed serological and/or molecular detection methods, provided molecular characterization of viral strains, or addressed zoonotic potential, food safety, and public health implications. Exclusion criteria comprised human case reports without a documented link and studies lacking relevant epidemiological, molecular, or ecological data. The collected data were compiled, critically evaluated, and integrated to provide a comprehensive overview of the role of wildlife in HEV epidemiology, as well as its emerging threats to human and animal health within the One Health perspective. Transmission pathways discussed in this review are inferred from epidemiological, ecological, and molecular evidence rather than being directly measured.

## 3. HEV Transmission Pathways and Global Distribution of Genotypes

The transmission of HEV occurs through multiple interconnected pathways that highlight the close relationship between humans, animals, and the environment. In Europe and other industrialized regions, zoonotic and foodborne routes predominate, with infections most often associated with the consumption of raw or undercooked pork and wild game meat, particularly from domestic pigs, wild boar, and deer [14,25]. These patterns underscore the growing recognition of wildlife as a central component in the epidemiological cycle of HEV. In contrast, in developing countries where HEV genotypes 1 and 2 are endemic, large outbreaks continue to occur mainly via fecal–oral transmission through contaminated water and inadequate sanitation [4,21]. Additional, though less common, transmission routes include vertical (mother-to-child) and iatrogenic pathways (e.g., blood transfusion), reported in both acute and chronic infections [2,3,6]. Environmental contamination also contributes substantially to transmission, as HEV shed in feces can persist in surface and groundwater, enabling long-term circulation among humans, animals, and ecosystems [17,32].

HEV genotypes are classified within the species *Paslahepevirus balayani* (formerly) and exhibit distinct host ranges and geographical patterns, ranging from strictly human (HEV-1 and HEV-2) to zoonotic (HEV-3 and HEV-4) and predominantly animal-associated genotypes (HEV-5 to HEV-8) [17,33]. Pronounced geographical variation has been observed in the distribution and circulation of HEV genotypes across both human and animal populations, reflecting differences in ecological conditions, host species, and food production systems [33]. These variations influence not only infection prevalence but also the dominant transmission pathways and the probability of zoonotic spillover. In Europe and Asia, zoonotic genotypes HEV-3 and HEV-4 account for most autochthonous infections in both humans and animals. HEV-3 predominates in Europe, where multiple subtypes (3a, 3b, 3c, 3e, 3f, 3h, 3i, 3j) have been reported in domestic and wild pigs [34]. The detection of these subtypes in deer and other wildlife species further supports evidence of interspecies transmission and underscores the ecological interconnectedness of HEV reservoirs [17]. Comparable findings have been reported in South America, where HEV-3 has been identified in domestic pigs, white-collared peccaries, and wild boar [35,36]. In Asian countries, both HEV-3 and HEV-4 have been detected in humans and animals, with particularly high seroprevalence among pig farmers and increased exposure risk for wild boar hunters [37,38]. Close and frequent human–animal contact in rural settings, together with traditional dietary practices involving pork consumption, facilitates bidirectional viral circulation and zoonotic spillover to humans [39]. In regions where HEV-3 and HEV-4 co-circulate, early nucleotide sequence analyses demonstrated a high degree of similarity between human and swine isolates, indicating a shared infectious source and supporting the concept of a unified epidemiological cycle [40,41]. The more recently identified genotypes HEV-5 and HEV-6 in wild boar, as well as HEV-7 and HEV-8 in camels, further extend the known host range of the virus [13,33]. Although these genotypes are primarily animal-associated, zoonotic transmission of HEV-7 to humans through the consumption of camel milk has been documented, illustrating the expanding ecological and epidemiological diversity of HEV [13]. A comparative overview across host species and geographical regions, together with molecular and serological prevalence data, is presented in Table 2.

**Figure 1:**
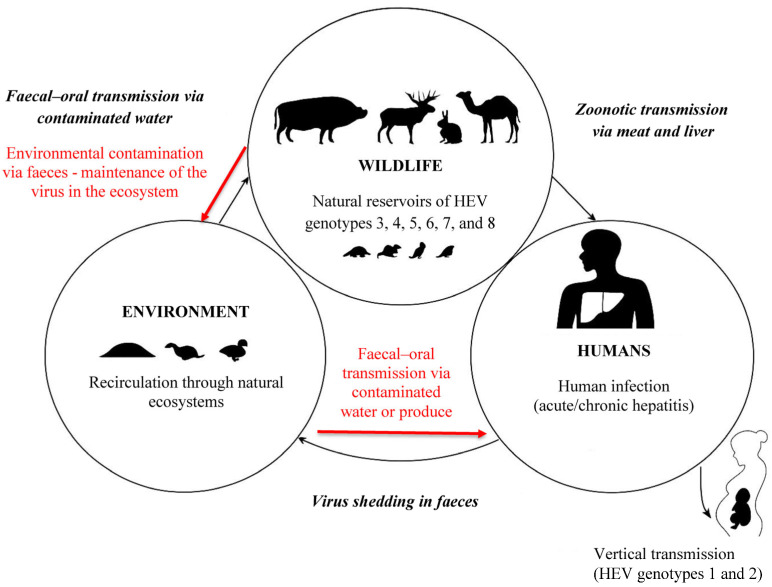
Transmission cycle of the hepatitis E virus. Legend (Geographical distribution of HEV genotypes): Genotypes 1 and 2—restricted to humans; prevalent in Asia, Africa, and Mexico. Genotype 3—zoonotic; widespread in Europe, North America, and parts of Asia. Genotype 4—zoonotic; mainly reported in East and Southeast Asia, recently also in Europe. Genotypes 5 and 6—zoonotic; identified in wild boars in Japan. Genotypes 7 and 8—zoonotic; detected in dromedary and Bactrian camels in the Middle East and Central Asia.

This interplay between humans, animals, and the environment underscores the necessity of a comprehensive One Health approach, integrating epidemiological surveillance, veterinary and public health control, and environmental monitoring. Such an integrated framework enables a more nuanced understanding of HEV ecology, improves the capacity to identify shared viral lineages circulating across wildlife, livestock, and humans, and strengthens evidence-based risk assessment [56], particularly regarding the contribution of wildlife to foodborne transmission and environmental exposure pathways (Figure 1).

## 4. HEV in Wildlife

Wildlife plays a pivotal role in the complex ecology of HEV, serving as both a reservoir and a conduit for zoonotic transmission cycles. While domestic pigs are recognized as the principal source of HEV-3 and HEV-4 infections in humans, accumulating evidence indicates that a wide range of wild species—particularly wild boar, deer, rabbits (*Leporidae*), and rodents—harbour genetically related viral strains of *Paslahepevirus balayani*, whereas rodents are primarily associated with *Rocahepevirus ratti* (formerly classified within *Orthohepevirus* C), a distinct hepevirus for which human infections have been reported only sporadically [23,30,31]. The detection of HEV RNA in liver, muscle, and fecal samples from these animals provides strong evidence of their role in sustaining viral circulation within natural ecosystems and contributing to potential spillover events. The increasing overlap between wildlife habitats and agricultural landscapes, coupled with heightened human exposure through hunting and consumption of game meat, further amplifies the risk of zoonotic transmission. Understanding HEV circulation among wildlife populations is therefore critical for evaluating its epidemiological impact and for guiding One Health-oriented surveillance and intervention strategies.

### 4.1. Epidemiological Trends and HEV-3 Prevalence in Wild Boar Populations Across Europe

Wild boars (*Sus scrofa*) are recognized as one of the most important natural reservoirs and significant carriers of the HEV worldwide. Over the past two decades, a notable increase in autochthonous cases of hepatitis E in humans has been observed, many of which have been directly linked to the consumption of raw or undercooked meat from domestic pigs, wild boars, and deer [25,45,57]. Globally, meta-analyses and large-scale seroepidemiological surveys indicate that the average seroprevalence of anti-HEV antibodies in wild boar populations is approximately 28% (95% CI: 23–34), while HEV RNA is detected in around 8% (95% CI: 6–10) of animals, confirming the widespread and persistent circulation of the virus in natural ecosystems [58]. Similar findings have been reported across multiple continents, demonstrating that HEV infection in wild boars is not restricted to specific ecological zones, but represents a global phenomenon affecting both free-living and farm-associated populations [58]. Early European studies reported HEV circulation in wild boar populations primarily on a serological basis, with an overall seroprevalence of approximately 10%, although HEV RNA was not detected in the same samples [59]. In contrast, more recent investigations conducted across various regions have documented substantially higher prevalence rates, typically ranging between 10% and 25%, reflecting temporal and spatial variability in viral circulation [25,30]. This high infection rate is particularly relevant in areas where pigs are raised under extensive or semi-intensive farming systems. In such settings, wild boars frequently come into direct contact with domestic pigs and may occasionally hybridize, facilitating viral transmission either through the fecal–oral route or via contact with the blood of infected animals. Molecular investigations have demonstrated a high degree of nucleotide sequence homology between HEV strains isolated from domestic pigs, wild boars, and humans, confirming a common infectious source and underscoring the zoonotic potential of the virus [30,60]. Experimental evidence further substantiates this interspecies link: domestic pigs cohabiting with HEV-infected wild boars became virus-positive within two weeks, with viral RNA detected in faeces, followed by seroconversion after four weeks [61]. Collectively, these findings reinforce the role of wild boars as a central epidemiological reservoir and a significant source of HEV transmission to domestic pigs and humans. In Spain, HEV was first detected in wild boars in 2008. Using an in-house ELISA on 150 serum samples collected from southern and central regions, a seroprevalence of 42.7% was reported, while HEV RNA was detected in 19.6% of samples by RT-PCR; sequence analysis confirmed that all isolates belonged to genotype 3 [48]. Subsequent investigations across the Iberian Peninsula demonstrated sustained circulation of HEV-3 in wild boar populations, accompanied by increasing molecular diversification. In particular, an emergent HEV-3 subtype was identified in wild boars from southwestern Spain [62], and later regional surveys reported variable seroprevalence and RNA detection rates, reflecting spatial and seasonal heterogeneity in viral circulation [25,62]. In Italy, early investigations detected HEV RNA in 25% of bile samples collected from hunted wild boars, with phylogenetic analyses revealing high similarity to European HEV-3 strains circulating in humans and domestic pigs [44]. These findings were subsequently confirmed by larger studies, reporting a seroprevalence of 56.2% and RNA detection in 9.4% of samples from central Italy, with all isolates classified as genotype 3 and closely related to human strains from the same region [47]. More recent surveillance has confirmed ongoing viral circulation, with HEV RNA detected in 10.2% of liver samples and 5.6% of muscle tissues from wild boars examined between 2019 and 2020, supporting persistent HEV-3 infection in free-living populations [30,45]. These observations were further substantiated by molecular investigations from south-eastern Italy, where HEV RNA was detected in wild boars and phylogenetic analyses demonstrated circulation of genotype 3 strains closely related to contemporary European lineages [63]. Recent European findings further highlight the food safety dimension associated with wild boar meat. Recent European studies have additionally highlighted the food safety dimension associated with wild boar meat. In central Italy, HEV RNA was detected in 1.35% of muscle tissue from hunted wild boars [64], while 10.87% of liver samples and 2.76% of diaphragm tissues tested positive in another investigation [45]. These findings indicate that HEV may be present in edible tissues entering the food chain, which is of particular concern in regions where traditional dishes involve raw or undercooked game meat [17,45,64]. Genomic surveillance has revealed marked genetic heterogeneity of HEV-3 circulating in European animal reservoirs. Recent studies in both domestic pigs and wild boars have identified multiple subtype clusters—including 3a, 3c, 3e, 3f, and the less common 3ra—across several European regions [30,65]. Wild boar populations in Central Italy, in particular, show pronounced subtype diversity, with the concurrent circulation of multiple HEV-3 lineages, indicating complex transmission patterns consistent with both sustained local circulation and possible external introductions. This subtype diversity reflects the dynamic evolutionary landscape of HEV-3 within wildlife reservoirs and supports the need for continuous molecular surveillance at the wildlife–livestock–human interface.

In Germany, HEV RNA was first detected in wild boars in the mid-1990s, preceding the widespread recognition of HEV as a zoonotic pathogen in Europe. Using multiple RT-PCR protocols, HEV RNA was found in 5.3% of 189 serum samples collected between 1995 and 1996, with phylogenetic analyses showing close genetic relatedness to strains from pigs and humans in the Netherlands and Japan [66]. More recent data confirm continued viral circulation: in a 2019 study conducted in north-eastern Germany, HEV RNA was detected in 33 of 393 wild boars [67], with all detected strains classified as genotype 3 in free-ranging populations. Comparable molecular evidence has also emerged from synurbic wild boar populations in Spain, with HEV-3 detected across multiple geographical regions in Europe [68]. In Slovakia, recent molecular studies detected HEV RNA in wild boars and other wildlife species, with phylogenetic analyses showing circulation of HEV-3 variants sharing high sequence similarity with strains identified in regional livestock and human infections [69]. Findings from Bulgaria revealed moderate-to-high seroprevalence in wild boars sampled in the western part of the country, compatible with endemic circulation [24]. In Portugal, targeted surveillance demonstrated a substantial HEV burden in wild boars and identified environmental and behavioural factors contributing to elevated zoonotic risks, particularly in areas where hunting and game meat consumption are common [25]. Historical data from Switzerland further support these observations, documenting significant seroprevalence in both wild boars and domestic pigs, reflecting ongoing circulation at the wildlife–livestock interface [70]. Molecular and epidemiological studies conducted over the past two decades have consistently confirmed the enzootic circulation of HEV-3 in Hungary and documented a close genetic relationship between strains identified in humans, domestic pigs, and environmental samples [71,72,73]. The role of wild boars in sustaining HEV-3 circulation within the Pannonian Basin was documented in an earlier report [74]. HEV has also been detected in wild boar in various countries across the Balkan Peninsula. In Croatia, a large-scale investigation of HEV seroprevalence in wild boar was conducted in 2016. Using a commercial ELISA test, 1000 serum samples collected from 16 counties were analysed. The overall seroprevalence reached 31.1%, with regional values ranging from 7.7% to 50.6%, and the highest prevalence was observed in the north-eastern part of the country, where wild boar density is highest. Real-time RT-PCR followed by confirmatory nested RT-PCR detected HEV RNA in 11.3% of seropositive animals. Most HEV-positive wild boars were younger than one year, indicating early-life infection and the potential for prolonged environmental shedding [29]. Given that HEV infection had already been confirmed in neighbouring countries such as Serbia and Hungary [74,75], the authors discussed the possibility of cross-border transmission associated with wild boar movement. Analyses of samples collected between 2010 and 2017 demonstrated endemic circulation of HEV genotype 3 in both wild boar and domestic pig populations, with seroprevalence in wild boars generally reported in the low single- to low double-digit range, whereas substantially higher seroprevalence, often exceeding 30%, was consistently observed in domestic pigs, indicating a sustained zoonotic exposure pathway for humans [76]. In Slovenia, a seroprevalence of 30.2% was reported among wild boars, while HEV RNA was detected in one of 288 analyzed serum samples [77]. Although molecular detection rates were low—likely due to the limited sensitivity of PCR testing on serum—the widespread presence of anti-HEV antibodies across multiple regions is consistent with prior exposure and ongoing circulation within wild boar populations. Comparable prevalence levels have been reported in neighbouring Central and South-East European countries, confirming that wild boars represent a key wildlife reservoir contributing to regional HEV epidemiology. In Serbia, the first investigations of HEV in wild boars were conducted in 2008, with no evidence of infection detected in the analyzed samples [78]. Subsequent investigations conducted by the same authors during the 2010/2011 hunting season, covering 27 hunting grounds across seven districts, revealed a seroprevalence of anti-HEV antibodies of 34.33%, while HEV RNA was detected in 9.4% of liver samples [79]. Further research [80,81] confirmed the widespread presence of HEV infection across wild boar populations nationwide. A study conducted in nine major hunting areas spanning northern, southern, western, eastern, and central Serbia showed an overall seroprevalence of 52.25%, with HEV-positive animals detected in all examined regions. The highest prevalence was recorded in the northern part of the country (Vojvodina), where 65.22% of wild boars tested seropositive. This regional distribution is consistent with patterns reported in neighbouring Central and South-East European countries. More recent findings further support the continuous circulation of HEV among wild and domestic pigs in Serbia. A study conducted on three commercial pig farms in the northern region reported a seroprevalence of 40.66%, reflecting continued circulation at the wildlife–livestock interface and underscoring the zoonotic risk associated with HEV genotype 3 in this region [82]. Taken together, available data confirm widespread circulation of HEV-3 in wild boar populations across Europe and its relevance within a One Health framework.

#### 4.1.1. Global Epidemiological Perspectives on HEV in Wild Boars Outside Europe

Although most knowledge of HEV-3 in wild boars comes from European studies, investigations in other parts of the world, particularly Asia and North America, also provide documented comparable patterns of HEV circulation, reservoir dynamics, and zoonotic transmission, thereby contributing to a more comprehensive understanding of associated public health risks. A longitudinal study in Northwest China documented sustained HEV circulation in both feral and farmed wild boars. In that study, HEV RNA was detected in liver, fecal, and serum samples, with RNA positivity ranging from 4.5% to 12% depending on age group and production system, while seroprevalence exceeded 35% [37]. Molecular characterization identified HEV genotype 4 as the predominant zoonotic genotype in East Asia. Although HEV-4 is not enzootic in Europe, this study demonstrates that free-ranging wild boars can maintain enzootic HEV transmission independently of domestic pig populations, mirroring ecological patterns observed in HEV-3 circulation within European ecosystems.

Additional evidence supporting the role of wild boars in global HEV epidemiology comes from earlier molecular investigations in East Asia, where HEV infections have been documented in free-ranging wild boar populations in Japan and Korea [83,84]. These studies reported combined serological and molecular prevalence levels between 20% and 50%, indicating that wild boars function as a stable wildlife reservoir capable of sustaining HEV circulation across diverse ecological and climatic settings. Although systematic surveillance of HEV in wild boar populations is largely absent in North America, several sources provide important contextual information regarding zoonotic risks in this region. A recent synthesis from the United States and Canada highlighted widespread HEV exposure in domestic pigs and frequent detection of HEV RNA in pork products, while emphasizing that data for free-ranging wild boars remain extremely limited [85,86]. A national risk assessment in Canada similarly identified HEV as a recognized foodborne hazard associated with pigs and pork and noted the lack of structured monitoring programmes for wild suid populations [87]. Public health authorities also acknowledge that undercooked meat from pigs, deer, and wild boars may represent a potential source of human infection, underscoring a recognized but insufficiently quantified zoonotic risk within the Canadian context [88]. In contrast to the well-characterized HEV-3 situation in Europe, available data from North America indicate widespread HEV exposure in pigs, while the specific genotype distribution remains poorly defined due to substantial surveillance gaps.

#### 4.1.2. Environmental and Ecological Drivers of HEV Circulation in Wildlife

Environmental and ecological factors have been recognized as key determinants of HEV-3 circulation in wild boar populations. Across Europe, wild boar numbers and geographic distribution have expanded considerably in recent decades, driven by increasingly mild winters and abundant natural and anthropogenic food resources [89,90]. These conditions enhance reproductive success, reduce overwinter mortality, and increase population density, thereby intensifying both intra-species interactions and interactions at the wildlife–livestock interface. EFSA assessments also highlight the critical role of ecological and management factors in shaping HEV exposure risks in European wildlife populations [17]. HEV is shed in substantial quantities in faeces, and numerous environmental monitoring studies have reported HEV RNA in sewage, surface waters, and other environmental matrices downstream of infected populations [91]. Although direct quantitative links between wild boar density and viral loads in soil or water remain limited, current evidence indicates that environmental contamination can act synergistically with direct and foodborne transmission routes, contributing to the persistence and spatial dissemination of HEV-3 within natural ecosystems [32,92]. Although the duration of HEV-3 shedding in wild boars remains insufficiently characterized, the frequent detection of HEV RNA in naturally infected animals suggests that viral excretion may extend beyond the acute phase, supporting enzootic maintenance [63]. Comparative evidence from Asia provides additional insight into ecological determinants of transmission. In China, high HEV prevalence has been observed in both feral and farmed wild boars, with substantially higher seroprevalence and fecal shedding in farmed populations, underscoring the influence of management practices, population density, and environmental exposure on viral maintenance [37]. These patterns mirror ecological dynamics in several European regions experiencing rapid wild boar population expansion. Taken together, current evidence shows that HEV-3 persistence in wild boar populations is shaped by interconnected ecological pressures, environmental contamination, prolonged shedding dynamics, and cross-species interactions. This complex, multifactorial landscape reinforces the integrated One Health framework summarized in Table 3.

### 4.2. HEV in Other Wildlife Species

Although wild boars are recognized as the main wildlife reservoir of HEV-3 in Europe, numerous studies have shown that other wild species may also harbour HEV or HEV-related variants. These species generally play a secondary or incidental epidemiological role; however, their involvement highlights the ecological diversity of HEV circulation and its ability to persist across heterogeneous wildlife communities.

**Cervids (deer species)**. HEV infection in cervids is now well documented across Europe, with multiple serological and molecular studies confirming the presence of HEV-3 or HEV-3-related strains in red deer (*Cervus elaphus*), roe deer (*Capreolus capreolus*), and fallow deer (*Dama dama*) [49,93,94,95]. Although infection levels in these species are generally lower than those observed in wild boars, recent phylogenetic analyses suggest that certain deer populations may act as true hosts for HEV-3 and HEV-4 rather than solely as incidental spillover species [95].

Reported seroprevalence in cervids varies considerably across Europe, usually ranging from 1 to 10%, with higher levels sometimes observed in regions where deer populations overlap ecologically with wild boars [93,94]. HEV RNA is detected much less frequently—most commonly in 1–3% of tested liver samples [49,93]. When viral RNA is present, sequence analyses consistently show close relatedness between deer-derived and wild boar-derived HEV-3 strains, indicating that infection in cervids mainly results from environmental exposure rather than independent transmission cycles [49,95]. Broader ecological reviews support these findings, suggesting that HEV circulation in cervids is generally sporadic and influenced by habitat use, landscape structure, and interactions with coexisting wild boar populations, rather than maintenance of the virus within deer populations alone [96].

Geographic patterns reinforce this interpretation. Recent studies from Japan demonstrate that cervids remain only sporadically infected with HEV, with extremely low seroprevalence and rare RNA detection in sika deer, while sympatric wild boars show substantially higher infection rates [83,97,98]. Similarly, surveys in northern and central Europe indicate that deer from regions with dense wild boar populations exhibit higher rates of exposure, whereas cervids inhabiting mountainous or low-contact environments show only sporadic seropositivity. For example, seroprevalence in Alpine chamois (*Rupicapra rupicapra*) and European red deer seldom exceeds 1–2%, and HEV RNA is typically undetectable in these populations [99]. Studies conducted in controlled or fenced hunting estates, where cervids have limited interaction with wild boars and are provided with controlled feed, report negligible risk, with no HEV RNA detected in farmed deer populations [100].

Taken together, current evidence indicates that cervids function mainly as incidental hosts and ecological sentinels: infection occurs sporadically, most often in landscapes characterized by environmental contamination with HEV shed by wild boars, but there is no indication that cervids sustain autonomous HEV transmission cycles. Their infection patterns, therefore, reflect ecosystem-level exposure rather than species-specific susceptibility and provide valuable insights into the spatial ecology of HEV circulation within wildlife communities.

**Lagomorphs (rabbits)**. Rabbits have received increasing attention as potential wildlife hosts of the hepatitis E virus (HEV) following the discovery of a distinct lineage, HEV-3ra, which clusters within genotype 3 but forms a genetically coherent variant predominantly associated with leporids. First detected in farmed rabbits in China and France, HEV-3ra has since been documented in both domestic and wild rabbit populations across Europe, Asia, North America, and Australia [50,51,52,101,102]. HEV RNA detection in wild rabbits is generally low—typically between 0.5% and 7%—yet sufficiently consistent to demonstrate that natural infection occurs within free-ranging lagomorph populations [51,52,103].

Phylogenetically, HEV-3ra strains show clear genomic divergence from classical HEV-3 subtypes circulating in suids. Full-genome analyses indicate that rabbit-derived HEV sequences form a distinct monophyletic clade, with nucleotide identities to HEV-3 strains typically below 80%, supporting their recognition as a leporid-associated sub lineage likely to have emerged through historical host-switching events [104].

In addition to circulation in wild rabbits, several studies in France, Germany, Spain, and China have detected HEV-3ra in farmed rabbits intended for human consumption, raising food safety concerns. Although zoonotic transmission appears rare, confirmed human infections with rabbit-derived HEV strains show that HEV-3ra can, under certain conditions, cross species barriers [105,106].

Despite these findings, rabbits are not considered significant contributors to HEV epidemiology in Europe. Prevalence is consistently lower than in wild boar, environmental viral loads associated with rabbits are minimal, and there is no evidence that rabbits maintain stable, independent HEV transmission cycles in the wild. Rabbits are therefore best regarded as occasional hosts harbouring a divergent HEV-3 variant with limited ecological impact, though with sufficient zoonotic relevance to warrant continued surveillance in regions where wild rabbits interact with swine or are consumed as game meat.

**Mesocarnivores. Mesocarnivores**—including red foxes (*Vulpes vulpes*), stone martens (*Martes foina*), badgers (*Meles meles*), raccoon dogs (*Nyctereutes procyonoides*), and wild felids—have been studied in multiple regions worldwide, yet consistent evidence shows that these species do not serve as true reservoirs of hepatitis E virus [96]. Across Europe, Asia, and North America, reported seroprevalence is generally very low (0–5%), and HEV RNA is only rarely detected in tissues or feces [57,96]. Phylogenetic analyses consistently show that viral sequences obtained from mesocarnivores cluster within HEV-3 or HEV-4 lineages circulating in sympatric suid populations, indicating that these infections represent incidental spillover rather than sustained intraspecies transmission [96].

Red foxes, which frequently inhabit peri-urban and agricultural landscapes and often scavenge on offal or carcasses, may test positive for HEV antibodies, but confirmed RNA detection remains exceptionally rare [26,53,96]. This supports the interpretation that foxes act primarily as environmental sentinels, reflecting localized contamination rather than functioning as competent HEV hosts. Similar patterns have been observed in martens and badgers: although these species may ingest contaminated tissues, no study has demonstrated sustained viral replication or evidence that mesocarnivores maintain HEV within their populations [26,96].

Current evidence indicates that mesocarnivores play a negligible epidemiological role in the ecology of HEV. Their infections are best interpreted as sporadic spillover events from suid reservoirs, while their occasional seropositivity may serve as an indicator of localized environmental contamination in areas where wild boars or domestic pigs are present. These observations align with broader One Health perspectives on HEV ecology, which emphasize cross-species spillover and environmental contamination as key drivers of viral circulation rather than sustained transmission cycles in non-suid wildlife hosts [56].

**Other wildlife species**. Beyond wild boars, cervids, lagomorphs, and mesocarnivores, several additional wildlife taxa are now recognized as hosts of genetically diverse *Hepeviridae*. Avian HEV, classified as *Orthohepevirus* B, is widely distributed in wild and domestic birds across multiple continents, where it causes hepatitis–splenomegaly syndrome in poultry but currently shows no confirmed zoonotic potential [107,108]. Rodent- and mustelid-associated viruses belonging to *Rocaherpesvirus ratti* exhibit broad host diversity: HEV-C1 (rat HEV) occurs in urban rodents and shrews, whereas HEV-C2 circulates in ferrets and mink [109,110]. Although HEV-C viruses are genetically distinct from zoonotic HEV-3 and HEV-4, sporadic human infections with rat HEV documented in Asia, Europe, and North America demonstrate that wildlife-associated strains can cross species barriers under specific circumstances [6,111,112]. Together, these findings illustrate that *Hepeviridae* can infect a broad spectrum of wildlife species, extending beyond classical reservoirs to various small mammals and avian taxa, thereby contributing to a wider pool of viral genetic and ecological diversity [113].

## 5. Synthesis and Perspectives

Growing evidence of *Hepeviridae* circulation in wildlife indicates that these infections should not be regarded as isolated findings but as integral components of a broader ecological system. Ecological changes, anthropogenic pressures, and interactions at wildlife–domestic–human interfaces influence opportunities for viral maintenance and potential spillover. From a One Health perspective, HEV in wildlife should be understood as part of a complex, interconnected ecological network in which it circulates. Land-use change, urban expansion, environmental contamination, water systems, domestic animal populations, and human activities interact to shape the conditions under which viral circulation and spillover occur. Therefore, integrating ecological and environmental data with veterinary and public health surveillance is essential for anticipating and mitigating zoonotic threats. Despite an expanding evidence base, substantial knowledge gaps remain. In most countries, HEV surveillance remains largely focused on domestic pigs and human clinical cases, while monitoring in wildlife is fragmented, inconsistent, or absent. At the EU level, EFSA recognizes the importance of wildlife reservoirs in the emergence of zoonotic pathogens and advocates a One Health-oriented approach to risk assessment; however, a harmonized and systematic surveillance framework specifically targeting HEV in wildlife has yet to be implemented. These limitations, together with limited molecular characterization of non-classical hosts, constrain the ability to evaluate transmission pathways, environmental contamination, and potential foodborne or occupational risks. Looking ahead, several research and surveillance priorities can be identified. Strengthening molecular surveillance in wildlife populations, particularly in species inhabiting peri-urban, agricultural, and human-modified environments, will be crucial for detecting emergent variants and assessing zoonotic potential. Long-term and longitudinal ecological studies are needed to clarify maintenance hosts, shedding dynamics, and environmental persistence. Improved integration of environmental, veterinary, and clinical data streams would facilitate earlier recognition of spillover events and support more effective risk assessment and response. In addition, targeted risk communication related to wildlife-associated HEV, especially concerning hunting practices, game meat handling, and rodent management, should be incorporated into broader zoonotic disease prevention strategies. Ultimately, sustained scientific investment, harmonized monitoring systems, and a strengthened One Health framework will be essential for understanding and managing the evolving public health relevance of hepatitis E virus at the wildlife–environment–human interface.

In conclusion, prioritizing coordinated wildlife surveillance and integrating it with existing public health and veterinary networks across Europe and worldwide will be key to mitigating zoonotic risks. Addressing current knowledge gaps and implementing harmonized monitoring strategies should be considered urgent priorities for policymakers and research institutions. Strengthening collaboration across sectors and borders will ultimately enhance countries’ capacity to anticipate, prevent, and respond to HEV emergence at the wildlife–environment–human interface.

## Figures and Tables

**Table 1:** Criteria applied in the literature selection process.

Criterion	Inclusion	Exclusion
Language	English or Serbian	Other languages without an English abstract
Type of publication	Original research, reviews, theses, technical or institutional reports	Editorials, letters, commentaries without data
Study period	2015–2025	Earlier publications, unless they provide foundational or seminal data on HEV ecology, host range, or transmission pathways
Study focus	HEV in wildlife species (e.g., wild boar, deer, rabbits, rodents, carnivores)	Human-only case reports or clinical studies without zoonotic context
Methods used	Serological and/or molecular HEV detection; molecular characterization; epidemiological relevance	Studies lacking clear methodological or analytical data

**Table 2:** Molecular and serological prevalence of hepatitis E virus (HEV) in wildlife host species across different geographical regions [42].

Host Species	Country/Region	Sample Type	Detection Method	Prevalence (%)	Reference
Wild boar (*Sus scrofa*)	Saxony, Germany	Serum	ELISA	233/960 (24.30)	[43]
		Internal organs	RT-PCR	5/960 (0.50)	
Wild boar (*Sus scrofa*)	Italy	Bile samples	RT-PCR	22/88 (25)	[44]
Wild boar (*Sus scrofa*)	Central Italy	Liver Muscle samples	RT-PCR	55/506 (10.87) 14/506 (2.76)	[45]
Wild boar (*Sus scrofa*)	Abruzzo, Italy	Liver, gallbladder	RT-PCR	11/116 (9.50)	[46]
Wild boar (*Sus scrofa*)	Central Italy	Blood Feces	ELISA RT-PCR	36/64 (56.20) 6/64 (9.40)	[47]
Wild boar (*Sus scrofa*)	Spain	Serum	ELISA RT-PCR	64/150 (42.70) 29/150 (19.60)	[48]
Wild boar (*Sus scrofa*)	Croatia	Serum	ELISA RT-PCR	311/1000 (31.10) 17/150 (11.33)	[29]
Roe deer (*Capreolus capreolus*)	Germany	Blood Liver	ELISA RT-PCR	0/59 (0) 5/78 (6.40)	[49]
Red deer (*Cervus elaphus)*	Germany	Blood Liver	ELISA RT-PCR	0/78 (0) 2/83 (2.40)	[49]
Fallow deer (*Dama dama*)	Germany	Blood Liver	ELISA RT-PCR	0/20 (0) 0/20 (0)	[49]
Wild rabbits (*Oryctolagus cuniculus*)	China	Serum	ELISA RT-PCR	191/335 (57) 25/335 (7.50)	[50]
Wild rabbits (*Oryctolagus cuniculus*)	Italy	Serum Liver	ELISA RT-PCR	14/35 (40) 4/35 (11.40)	[51]
	United Kingdom	Serum Liver	ELISA RT-PCR	2/30 (6.70) 2/30 (6.70)	
Wild rabbits (*Oryctolagus cuniculus*)	Portugal	Serum Feces	ELISA RT-PCR	5/205 (2.44) 0/120 (0)	[52]
Raccoons (*Procyon lotor*)	Brandenburg, Germany	Body cavity transudate	ELISA RT-PCR	25/73 (34.30) 0/73 (0)	[26]
Red fox (*Vulpes vulpes*)	Croatia	Muscle extracts Feces	ELISA RT-PCR	0/692 (0) 0/692 (0)	[53]
Jackal (*Canis aureus moreoticus*)	Croatia	Muscle extracts Feces	ELISA RT-PCR	0/171 (0) 0/171 (0)	[53]
Wolf (*Canis lupus*)	Italy	Rectal swabs	RT-PCR	1/42 (2.30)	[54]
Red fox (*Vulpes vulpes*)	Italy	Serum	ELISA	0/18 (0)	[55]
Wolf (*Canis lupus*)		Serum	ELISA	1/4 (25)	
Badgers (*Meles meles*)		Serum	ELISA	0/4 (0)	
Hedgehog (*Erinaceus europaeus*)		Serum	ELISA	0/8 (0)	
Porcupines (*Hystrix cristata*)		Serum	ELISA	0/5 (0)	
Roe deer *(Capreolus capreolus)*		Serum	ELISA	1/3 (33.33)	

Abbreviations: ELISA, enzyme-linked immunosorbent assay; RT-PCR, reverse transcription polymerase chain reaction.

**Table 3:** One Health framework of HEV-3 epidemiology in wild boars in Europe and associated zoonotic risks.

One Health Component	Key Elements	Evidence from Wildlife Studies
Wild boar reservoir	High HEV-3 prevalence; enzootic circulation; prolonged viral shedding, particularly in juveniles	Seroprevalence commonly reported at 20–60%; HEV RNA frequently detected in liver, muscle, bile and feces; shedding reported for several weeks.
Transmission pathways	Wildlife–livestock interface; environmental contamination; foodborne exposure	High genetic similarity between wild boar, pig and human strains; HEV RNA detected in soil, surface water and sewage; infectious virus identified in edible tissues.
Ecological and environmental drivers	Population expansion; habitat overlap; climate-related factors; anthropogenic food sources	Increasing wild boar density across Europe; mild winters and abundant food resources associated with enhanced population growth and transmission opportunities.
Viral circulation and evolution	Co-circulation of multiple HEV-3 subtypes; cross-species transmission	Subtypes 3a, 3c, 3e, 3f and 3ra reported in wild boars and pigs; regional clustering with evidence of repeated viral introductions.
One Health implications	Food safety risks; zoonotic transmission; need for integrated surveillance	HEV RNA detected in muscle, liver and diaphragm; traditional consumption of raw/undercooked game meat; relevance of coordinated wildlife–livestock–human monitoring

## Data Availability

No new data were created or analyzed in this study. Data sharing is not applicable to this article.
